# Colorectal cancer literacy and related factors in northeast of Iran: A cross‐sectional study

**DOI:** 10.1002/cnr2.2037

**Published:** 2024-03-24

**Authors:** Mohammad Hossein Delshad, Fatemeh Pourhaji, Mahbubeh Abdollahi, Hajar Pardeh Khorram, Atefeh Pourhasan

**Affiliations:** ^1^ Department of Public Health Department Torbat Heydariyeh University of Medical Sciences Torbat Heydariyeh Iran; ^2^ Health Sciences Research Center Torbat Heydariyeh University of Medical Sciences Torbat Heydariyeh Iran; ^3^ Social Determinants of Health Research Center Torbat Heydariyeh University of Medical Sciences Torbat Heydariyeh Iran; ^4^ Department of Public Health, School of Health Torbat Heydariyeh of Medical Sciences Torbat Heydariyeh Iran

**Keywords:** attitude, colorectal cancer, health literacy, knowledge

## Abstract

**Background:**

Colorectal cancer (CRC) is a health challenge and the second most common cancer worldwide. Increasing colorectal cancer literacy (CRCL) is one of the most effective factors in CRC prevention.

**Aim:**

The aim of this study was to determine and evaluate CRCL and its related factors in Torbat Heydarieh, northeastern Iran.

**Methods and Results:**

This study was a cross‐sectional survey conducted in 2021 in Torbat Heydarieh, a city in northeastern Iran, on 200 clients presenting to a comprehensive health service centers.

In addition to collecting sociodemographic characteristics, participants were administered the Knowledge and Attitude Questionnaire and the Colorectal Cancer Literacy Questionnaire (CRCLQ).

Data were analyzed with SPSS software version 25 using independent samples *t*‐tests, one‐way analysis, chi‐square, and Pearson correlation coefficients. The statistical significance level was set at *p* < .05. The results showed that the mean age of the participants was 51.12 ± 8.45 years. The majority of participants (84%) stated that their friends and relatives had no history of CRC.

Pearson correlation coefficient results showed a significant correlation between knowledge and attitude toward CRC (*r* = .15, *p* = .041). A significant correlation was also found between knowledge and CRCL (*r* = .4, *p* ≤ .001).

**Conclusion:**

We found low CRCL among clients of comprehensive health service centers. More targeted educational interventions are needed to promote CRCL among Iranian adults.

## 1 INTRODUCTION

Colorectal cancer (CRC) is a health challenge and the second most common cancer worldwide.^1^

According to the study Global CRC burden in 2020 and projections to 2040, approximately 1.9 million people were diagnosed with CRC and 0.9% death cases.^2^ The research reported deaths caused by were 515 637 among males and 419 563 among females, respectively.^2^

Similarly, CRC is the most common cancer among Iranian population in 2015 and the incidence and mortality rates of CRC were 9.0% and 7.4%, respectively.^3^ In addition the incidence of CRC among Iranian men and women was 5.56 and 4.30 per 100 000, respectively.^3^ According to the National and subnational Burden of Diseases (NASBAD), the trend of CRC showed that the incidence rate of CRC in Razavi Khorasan province was 7.72 per 100 000 for females and 7.06 per 100 000 for males.^4^ The rising trend in CRC therefore makes it the more important to take preventive measures and identify the prognostic factors.^5^

The studies indicate that 70%–80% of CRC cases are caused by environmental factors and lifestyle, such as diet (low intake of fruits and vegetables, daily alcohol consumption, and high intake red meat), smoking, lack of physical activity,^6^ family history, unfavorable socioeconomic status, obesity, preparation methods,^7^ abdominal obesity, hormone replacement therapy, high egg consumption, and nonsteroidal anti‐inflammatory drugs.^8^ Risk factors influencing CRC include colon polyps, inflammatory bowel disease, and ulcerative colitis in particular.^8^

Raising awareness and CRC screening play an important role in promoting and health healthy lifestyle and can help to further reduce CRC morbidity and mortality.^2^

Some studies have shown that health literacy (HL) is an important factor influencing client participation in CRC screening.^9^

HL involves the ability to obtain, read, and interpret health‐related information in order to make the necessary decisions about one's own health status.^10^

Some studies report that differences in HL lead to health disparities,^11^ decreasing health outcomes,^12^ and increase healthcare costs.^13^ In addition, people with lower HL are less likely to use preventive services^14^ and specially screening programs.^15^ Furthermore, important key associations indicated that more research is needed to better understand the relationship between HL and outcomes in order to recognize the negative impact of at‐risk populations and low HL. A multidimensional and comprehensive assessment of HL that assesses reading and listening skills is a solid foundation for such efforts.^16^

Due to the importance of this deadly cancer in the world, various studies have been conducted to estimate the burden of this disease and the cost it imposes on society.^17^, ^18^ Given the increasing prevalence of CRC and the low‐survival rate of patients due to advanced diagnosis, cancer was also recognized as a potential problem in Iran, and screening programs were introduced at national level to reduce the incidence.^19^

One of the effective factors for the prevention of CRC is the promotion of HL. Low HL should be considered a challenge not only for those affected but also for health care providers and health care systems due to lack of health care, preventive measures, and cancer screening.^20^

Lack of literacy in cancer knowledge and preventive behaviors are the main barriers to cancer screening and empowerment. They can influence cancer screening, diagnosis, and consequences.^10^ Cancer fear and risk perception are the most important predictors of cancer prevention programs and interventions.^21^ Studies have shown that low‐economic status, poor health, declining social support, and low HL are associated with a lower participation in CRC screening.^22^, ^23^ Therefore, HL and individual empowerment can be important determinants factors on the rate of CRC screening.^24^, ^25^

Empowerment is a positive, dynamic, and multidimensional concept in cancer prevention and treatment^26^ and helps to encourage people to undergo cancer screening. The aim of this study was to determine and evaluate CRCL and its related factors in Torbat Heydarieh, northeastern Iran.

## 2 METHODS

This study was a cross‐sectional survey conducted among 200 clients referring comprehensive health centers in the city of Torbat Heydarieh in northeastern Iran in 2021. Sampling was done in clusters, that is 19 health centers were considered as clusters and samples were selected in equal proportions.

In addition to recording sociodemographic characteristics, participants were administered the Knowledge and Attitude Questionnaire and the Colorectal Cancer Literacy Questionnaire (CRCLQ).

## 3 MEASURES

The research instrument included sociodemographic characteristics, such as age, gender, marital status, education level, occupational status, and family history of CRC, the knowledge and attitude questionnaire, and the researcher‐made CRCLQ.

The knowledge questionnaire contained 10‐item about CRC, etiology, awareness of diagnosis test of CRC, such as fecal occult blood testing (FOBT), flexible sigmoidoscopy, and colonoscopy. The responses were scored on using a three‐point scale (1 = “True,” 0 = “False,” 0 = “Uncertain”). ^27^ The scores of all 10 items are added together to obtain the total score; the range for the total score is therefore 0–10.

The attitude questionnaire contained 10‐item on whether screening tests are perceived as harmful or painful, whether they are inconvenient or expensive and how worried people are that cancer could be detected during screening. The answers are rated on a five‐point scale (5 = “extremely,” 4 = “very,” 3 = “somewhat,” 2 = “a little,” 1 = “not at all”). ^27^

The CRCLQ is a 33‐item scale, designed to measure CRCL among participants. The dimensions of the CRCLQ include understanding (7 items), access (4 items), reading (2 items), appraisal (4 items), and decision (6 items). The scores of all 33‐item are summed to obtain the total score; thus, the range for the total score is 33–165. Higher scores indicate greater CRCL.

Face and content validity of the CRCLQ was ensured by an expert panel (*N* = 10) consisting of six health education experts, three oncologists, and one psychologist. They assessed the relevance of the item to the local culture and clarity, which together from the content validity ratio (CVR) items. The necessity of an item was assessed using the CVR, and items with a score <0.62 were deleted according to Lawsh's.^28^ CVR was calculated 0.81. The experts were also asked to determine the simplicity, relevance, and clarity of the items and calculated content validity index (CVI). The CVI was estimated 0.79, which is considered satisfactory.^29^

The research instrument was completed by 30 participants and the internal consistency of the structures was determined by calculating Cronbach's alpha coefficient.^29^

The internal consistency of the questionnaire was determined by calculating Cronbach's alpha coefficient.^29^ The Cronbach's alpha was calculated for the knowledge questionnaire (*α* = 0.79), the attitude questionnaire (*α* = 0.80), and CRCLQ (0.78).

The data were analyzed through SPSS software version 25, using independent samples *t*‐tests, one‐way analysis, chi‐square analysis, and Spearman correlation. The statistical significance level was set at *p* < .05.

## 4 ETHICAL CONSIDERATION

This study was conducted in accordance with the Declaration of Helsinki and was approved by the Ethics Committee of Torbat Heydariyeh University of Medical Sciences (Approval ID: IR. THUMS.REC.1400.023). First, the participants were informed about objectives of the study and their written informed consent was obtained.

## 5 RESULTS

The majority of the 168 participants (84%) stated that their friends and relatives had no history of CRC. The results showed that the participants' mean age was 51.12 ± 8.45 years. More than half of them were married (64%), and approximately 51.5% of the participants were higher education (Table 1).

**TABLE 1:** Participant characteristics (*n* = 200).

Variable	Categories	*N* (%)
Age (years)	Mean ± SD: 51.12 ± 8.45	
Gender	Male	90 (45)
	Female	110 (55)
Marital status	Married	128 (64)
	Single	72 (36)
Education level	Middle and lower	44 (22)
	High school and diploma	53 (26.5)
	Higher education	103 (10351.5)
Occupational status	Housewife	73 (36.5)
	Employed	37 (18.5)
	Unemployed	48 (24)
	Self‐employment	42 (21)
Income^a^ Currency: Rial/Frequency: Monthly	≤10 million	16 (8)
	10–30 million	18 (9)
	30–50 million	10 (5)
	50–70 million	76 (38)
	70–100 million	58 (29)
	100–150 million	22 (11)
History of CRC cancer in family	Yes	32 (16)
	No	168 (84)
Bleeding in the lower gastrointestinal during the last month	Yes	18 (9)
	No	173 (86.5)
	I do not know	9 (4.5)
Constipation during the last month	Yes	83 (41.5)
	No	114 (57)
	I do not know	3 (1.5)
Positive medical history, abdominal pain, diarrhea, and feeling of fullness in the anus after defecation in the last month	Yes	62 (31)
	No	130 (65)
	I do not know	8 (4)
Loss of more than 10% of body weight in the last 6 months	Yes	24 (12)
	No	176 (88)
Having a history of CRC	Yes	26 (13)
	No	174 (87)

^a^ Number of households.

The results showed that the mean scores for knowledge, attitude, and CRCL were 2.88 ± 2.83, 27.24 ± 3.91, and 50.33 ± 10.17, respectively.

Spearman correlation results showed that there was no significant relationship between age and knowledge (*p* = .92) and attitude (*p* = .08). However, Spearman correlation showed that CRCL was correlated with age (*p* = .04).

Independent_samples *t*‐tests revealed that there was no significant relationship between gender and knowledge (*p* = .68), attitude (*p* = .71), and CRCL (*p* = .38). Independent_samples *t*‐tests also showed that there was no significant relationship between marital status and knowledge (*p* = .72) and CRCL (*p* = .41). However, this test showed that attitude was correlated with marital status (*p* = .03).

The findings of the ANOVA test showed that the participants with a higher level of education had the highest level of knowledge (*p* = .004) and CRCL (*p* < .001).

The results of the ANOVA test revealed that employee participants showed statistically higher levels of knowledge, and CRCL compared with the other participants (*p* < .001). However, the ANOVA test indicated there was no correlation between attitude and occupational status (*p* = .65) (Table 2).

**TABLE 2:** Sociodemographic characteristics and the mean of knowledge, attitude, and colorectal cancer literacy based on sociodemographic characteristics.

Variables	Knowledge mean ± SD	*p* value	Attitude mean ± SD	*p* value	Colorectal cancer literacy mean ± SD	*p* value
Gender		.68^a^		.71^a^		.38^a^
Male	2.53 ± 2.97		26.50 ± 3.79		47.71 ± 8.86	
Female	2.37 ± 2.58		26.28 ± 4.03		46.13 ± 8.74	
Marital status		.72^a^		.03^a^		.41^a^
Single	2.93 ± 2.97		27.70 ± 3.79		49.91 ± 10.86	
Married	2.77 ± 2.58		26.38 ± 4.03		51.13 ± 8.74	
Educational level		.004^b^		.81^b^		<.001^a^
Middle and lower	1.80 ± 2.14		27.323 ± 3.60		45.05 ± 9.15	
High school and diploma	2.58 ± 2.74		27.64 ± 3.03		48.79 ± 10.06	
Higher education	3.43 ± 2.30		27.13 ± 4.29		53.08 ± 9.70	
Occupational status		<.001^a^		.65^b^		<.001^a^
Household	2.34 ± 2.64		27.34 ± 3.54		45.53 ± 9.79	
Employee	4.59 ± 3.05		27.86 ± 4.16		53.73 ± 10.81	
Unemployed	3.31 ± 2.68		26.81 ± 4.18		52.75 ± 9.61	
Self‐employment	1.55 ± 2.18		27.07 ± 3.88		50.62 ± 8.63	
Income		<.02^b^		.19^b^		.99^b^
≤10 million	1.18 ± 1.18		25.12 ± 2.82		50.31 ± 7.26	
10–30 million	2.94 ± 3.03		27.61 ± 3.29		49.38 ± 11.74	
30–50 million	2.60 ± 3.23		27.10 ± 3.63		49.20 ± 5.55	
50–70 million	3.10 ± 3.04		27.81 ± 3.87		50.09 ± 10.33	
70–100 million	3.18 ± 2.64		27.50 ± 4.25		50.85 ± 10.69	
100–150 million	1.45 ± 2.13		26.50 ± 4.18		50.77 ± 10.52	

^a^ Independent *t*_ test.

^b^ One‐way ANOVA.

The results of bivariate associations based on Pearson correlation coefficient showed there was a positive and significant correlation between knowledge and attitude about CRC (*r* = .15, *p* = .041). Also, the finding indicated there was a positive and significant correlation between knowledge and CRCL (*r* = 0.4, *p* ≤ .001) (Table 3).

**TABLE 3:** Correlation among knowledge, attitude, and colorectal cancer literacy (CRCL).

Scale	Knowledge	Attitude	CRCL
Knowledge	‐		
Attitude	*r* = .15^a^	‐	
	* *p* = .041		
CRCL	*r* = .4^a^	*r* = .13^a^	‐
	* *p* ≤ .001	*p* = .09	

^a^ Pearson correlation coefficient.

* *p* ≤ .001.

The multiple linear regression models showed educational level (*p* < .001) was significant associations of knowledge, so that the best predictor of knowledge educational level. The multiple linear regression models showed marital status (*p* < .001) was significant associations of attitude, so that marital status was the best predictor of attitude. This test indicated that educational level (*p* < .001) was significant associations of CRCL, so that the best predictor of CRCL educational level (Table 4).

**TABLE 4:** Multiple linear regression model of knowledge, attitude, and colorectal cancer literacy (CRCL) with sociodemographic characteristics.

Variables	Knowledge			Attitude			CRCL		
	B	Std. error	*p* value^a^	B	Std. error	*p* value^a^	B	Std. error	*p* value^a^
Age	−0.028	0.019	.13	−0.006	0.027	.82	−0.065	0.066	.32
Marital status	0.251	0.327	.44	1.370	0.465	<.001	0.513	1.147	.60
Educational level	0.329	0.095	<.001	0.032	0.135	.81	1.565	0.333	<.001
Income	0.038	0.040	.80	0.089	0.211	.67	−0.108	0.522	.83
Occupational status	0.017	0.142	.90	0.091	0.201	.65	0.677	0.497	.17

^a^ Multiple linear regression.

## 6 DISCUSSION

The results of the present study provided some insights into the association between the knowledge, attitudes, and CRCL. The findings also indicated that some socioeconomic characteristics were predictive factors of knowledge, attitudes, and CRCL among the clients of comprehensive health centers in Torbat Heydarieh city.

The results showed that the level of CRCL was low. Then, it seems that the reasons for the low status of CRCL to provide good evidence for program decision makers in designing ongoing and future health interventions. Our results show that most participants had low knowledge of CRC screening behavior. This was related to the socioeconomic status.

The findings also indicated that some socioeconomic characteristics were predictive factors of knowledge and CRCL, so that higher levels of education had statistically higher levels of knowledge and CRCL. The results showed that marital status was a predictive factor for attitude and female participants had better attitude. Likewise, another study revealed that lack of CRC screening is related with low‐socioeconomic status.^30^, ^31^

The results showed that was no significant relationship between age and knowledge and attitude, respectively. Consistently, previous study reported similar results.^32^

According to the present study, there was a significant association between knowledge and educational level. This finding was in contradiction with study of Christou et al.^32^ and consistent with Al‐Thafar et al.^33^

The results showed no association between CRCL and attitudes. Inconsistently, a study suggested that low HL is associated with negative attitudes toward CRC screening.^34^

In the current study, only a small proportion of participants (3%) had at least one prior history of CRC screening compared with studies from Hong Kong (14%), in Korean (>45%),^35^ USA (45%).^36^ This difference may be due to the fact that colonoscopies in Iran are performed by opportunistic screening. Furthermore, they are dependent on people who voluntarily apply and bear the costs. Therefore, it is necessary that the public population to educate about the importance and necessity of CRC screening.

Notably, most participants did not perform a colonoscopy, possibly due to inappropriate HL. Awareness of screening or other reasons that may have limited their access to screening.^25^

Therefore, it is very important to discover the significant barriers to colonoscopy screening among the Iranian population. A significant number of individuals remain under‐screened due to the fears of cost and availability of screening, lack of health insurance, screening‐related pain, positive findings, and fear of a poor outcome during screening.^37^

Looking at the present study, higher knowledge was associated with better CRCL. Obviously, CRCL can influence an individual's decision about whether to see a doctor in time and improvement tendency to perform screening tests.

The research indicated that adequate HL about CRC was related to screening and that the level of HL was likely to be important.^10^, ^32^

The incidence rate of CRC is high in Iran. Therefore, there is a need to focus on HL in screening programs and using scientific and evidence‐based data as a basis for measuring the effectiveness of further health promotion campaigns.

Based on our results, high‐risk individuals had insufficient knowledge. In addition, they were not aware of cancer risk factors and regular screening.

To improve HL several strategies could be applied. First of all, the government should learn from the initiatives of different countries in this area. The second would be improving the healthcare providers' ability and skills in consulting with targeted populations and performing effective training national campaigns using the internet, web, and other methods as educational tools for the scientific dissemination of CRC prevention information. In addition, health promotion campaigns should be focused on those who do not visit their doctor regularly and do not have the CRC disease, and those who have less HL.

Also, strengthening the primary care level and covering the cost of screening by health insurance companies. In addition, establishing CRC screening program into the health care system in the future.^25^, ^38^ Noticeably, further research is needed to explore other aspects of CRCL and effective intervention to increase it.

## 7 LIMITATIONS

The strength of this study is that it measured CRCL and related factors among clients referring comprehensive health service centers in Torbat Heydarieh city of Northeast Iran for the first time. This is a strength because the relationship between CRCL and related factors among clients has been somewhat understudied. This study had several limitations. Participants were recruited clients health centers. Therefore, they are likely to be more spiriting in their health‐related behavior, better informed about health issues and have a larger disposal to prevention messages.

## 8 CONCLUSION

We found a low CRCL among the clients of comprehensive health service centers. More aimed educational interventions are needed to promote Iranian adults' CRCL.

## AUTHOR CONTRIBUTIONS

**Mohammad Hossein Delshad:** Conceptualization (equal); project administration (equal); resources (equal); validation (equal); visualization (equal); writing – original draft (equal); writing – review and editing (equal). **Fatemeh Pourhaji:** Conceptualization (equal); data curation (equal); formal analysis (equal); funding acquisition (equal); investigation (equal); methodology (equal); project administration (equal); resources (equal); software (equal); supervision (equal); validation (equal); visualization (equal); writing – original draft (equal); writing – review and editing (equal). **Mahbubeh Abdollahi:** Data curation (equal); software (equal). **Hajar Pardeh Khorram:** Data curation (equal); validation (equal). **Atefeh Pourhasan:** Conceptualization (equal); methodology (equal); resources (equal).

## CONFLICT OF INTEREST STATEMENT

The authors have stated explicitly that there are no conflicts of interest in connection with this article.

## ETHICS STATEMENT

This study was conducted by the Ethics Committee of Torbat Heydariyeh University of Medical Sciences (ID:IR.THUMS.REC.1400.023). First, the aims of the study were explained to the participants and their written informed consent was obtained from participants.

## Data Availability

Availability of Data and Materials Data are available from the corresponding author upon reasonable request.
